# TRPV1 and thermosensitivity

**DOI:** 10.1016/j.jphyss.2025.100009

**Published:** 2025-02-01

**Authors:** Makoto Tominaga, Moe Iwata

**Affiliations:** Thermal Biology Research Group, Nagoya Advanced Research and Development Center, Nagoya City University, Nagoya 467–8601, Japan

**Keywords:** TRPV1, Temperature, Thermosensitivity, Ion channel, Noxious heat detection

## Abstract

The capsaicin receptor TRPV1 was identified as the first heat-activated ion channel in 1997. Since then, numerous studies have been performed on its physiological functions and structure-function relationship, and chemicals targeting TRPV1 have been developed. It has been more than 27 years since the initial cloning of the *TRPV1* gene and more than 11 years since the clarification of its structure at the atomic level using cryo-EM. However, we still lack good chemical antagonists of TRPV1 as medicines. TRPV1 is involved in body temperature regulation, but how TRPV1 antagonists cause hyperthermia and how TRPV1 is involved in body temperature regulation are not yet clearly understood. More research is needed in the thermal biology field.

## 1 Introduction

A lot of papers have been published on TRPV1, from its physiological function and structural analysis to its role in human diseases. However, relatively few studies, especially reviews, have been conducted on its role in temperature sensitivity. Thus, this review specifically focuses on TRPV1 and thermosensitivity.

## 2 Cloning of the TRPV1 gene

Although the concept of temperature-dependent activation of ion channels was proposed long ago, TRPV1 was the first ion channel clearly shown to be activated by temperature changes. The receptor for capsaicin was reported as an ion channel in 1996, in which a study showed that capsaicin activates a nonselective cation channel with high Ca^2+^ permeability in the dorsal root ganglion (DRG) neurons of rats [1]. This discovery eventually led to identification of the gene encoding the capsaicin receptor, with research groups competing to clone this gene.

**Fig. 1:**
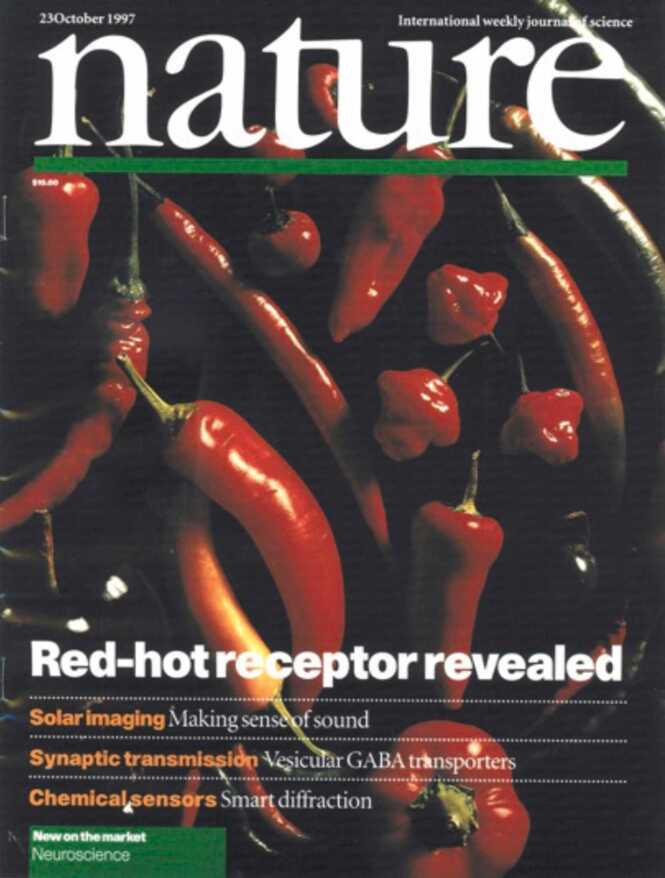
Cover art from a *Nature* journal issue of the cloning of the capsaicin receptor TRPV1 in 1997.

The lab of David Julius identified a capsaicin receptor gene using a Ca^2+^-imaging-based expression cloning method (Fig. 1) [2]. Specifically, they created a cDNA library from rat and mouse DRG neurons containing approximately 16,000 cDNAs. They searched for cDNA pools in the library that cause increase in the cytosolic Ca^2+^ concentrations upon transfection into HEK293 cells after application of capsaicin. And then, they eventually isolated a gene encoding a capsaicin receptor and named it vanilloid receptor subtype 1 (VR1), due to the vanilloid moiety in the structure of capsaicin. Later, the name of VR1 was changed to transient receptor potential vanilloid 1 (TRPV1), constituting the first member of the TRPV subfamily.

**Fig. 2:**
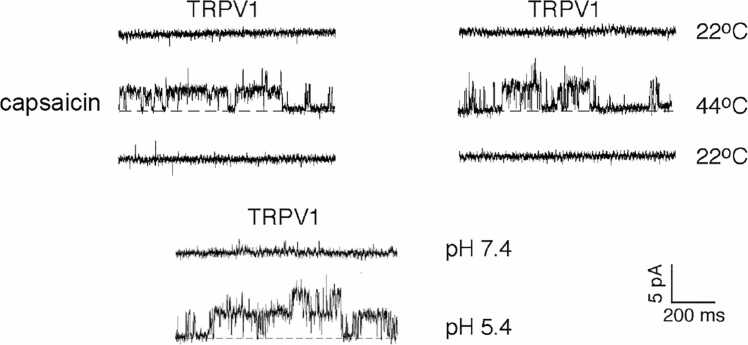
Single-channel recordings of rat TRPV1 activated by three different stimuli in membrane patches excised from HEK293 cells. (modified from Tominaga et al., 1998).

Because we feel hot sensation when we eat pungent capsicums, the Julius lab applied heat stimulation to TRPV1-expressing HEK293 cells. It was found that TRPV1 was activated by application of a pre-heated solution, and that the threshold temperature was above 43 °C, which is the threshold temperature causing pain sensation in humans and monkeys; this indicated that TRPV1 is not a simple heat receptor but a receptor for noxious heat stimuli. In addition, they observed flickered opening of single channels in the excised membrane patches upon heat stimulation (Fig. 2) [3], suggesting that TRPV1 is activated by heat directly without involvement of any cytosolic components. Direct heat-evoked activation of TRPV1 was proven later by recordings from TRPV1-containing soybean proteoliposomes in response to a temperature ramp (22–48 °C) [4].

## 3 Characterization of the heat-sensing ability of TRPV1

**Fig. 3:**
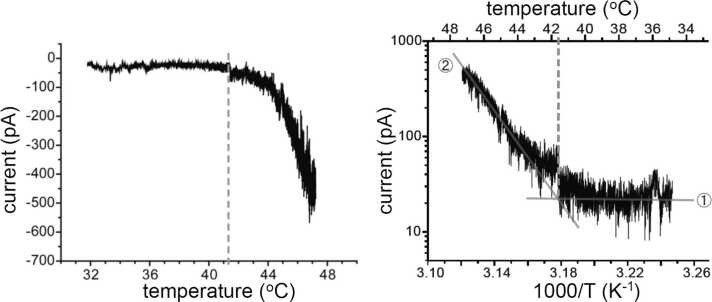
Whole-cell currents in HEK293 cells expressing rat TRPV1 (left) and an Arrhenius plot (right) from the left trace. The temperature threshold is determined by the intersecting point from the two straight lines before activation (1) and after activation (2).

Are the temperature thresholds stable? Julius et al. found synergism among the effective stimuli for TRPV1. For example, in the presence of stimuli other than heat such as capsaicin and acid, the temperature thresholds were reduced to below 43 °C [3]. In addition, post-translational modifications such as PKC-dependent phosphorylation reduce temperature thresholds further, and TRPV1 is activated by the temperatures within our body temperature range, explaining the spontaneous pain sensation [5], [6]. In many cases, Arrhenius plots have been used to determine temperature thresholds, which correspond to the intersecting points in the two lines with different slopes (Fig. 3) [4]. Furthermore, Q_10_ values greater than 10 (∼30) were also calculated from heat-evoked channel activation, while most other ion channels show Q_10_ values of 2–3, generally from the increase in Brownian motion of permeant ions [7]. Such properties of TRPV1 in HEK293 cells well resemble the heat-activated currents in cultured DRG neurons from neonatal rats [8].

Although there are few reports of single-channel recordings of TRPV1 [9], raising the temperature rapidly increases the opening frequency. Despite the large temperature coefficient of the apparent activity (Q_10_ ∼27), the unitary current, the open dwell times and the intraburst closures were only weakly temperature dependent (Q_10_ < 2). Instead, heat had a localized effect on the reduction of long closures between bursts (Q_10_ ∼7) and the elongation of the burst duration (Q_10_ ∼32), a unique property of heat-evoked activation, supporting the concept that heat stimulation has a distinct gating mechanism for TRPV1 activation.

## 4 Knockout mouse phenotype

The phenotypes of TRPV1-knockout (TRPV1KO) mice were reported by the Julius group in 2000 [10]. TRPV1KO mice showed significantly longer withdrawal latency at temperatures > 50 °C compared to wild-type (WT) mice, but no differences at 46 °C and 48 °C in the tail immersion test, although the temperature threshold for TRPV1 was ∼43 °C in vitro [2], [3]. This may be explained by two possible reasons: a difference in temperature between the mouse foot skin surface and sensory nerve endings, and the existence of thermosensing molecules other than TRPV1. The latter reason turned out to be the case, because it was reported that TRPA1 and TRPM3 are involved in the detection of noxious heat stimuli in addition to TRPV1 based on the result that mice lacking TRPV1, TRPM3 and TRPA1 (triple knockout mice) completely lost their response to noxious heat stimuli [11]. Now, it is understood that all the three TRP channels are involved in noxious heat sensing, which is consistent with the finding that TRPA1 has been a heat sensor throughout evolution [12].

TRPV1KO mice exhibited no obvious changes in circadian body temperature fluctuations or tolerance to increased (35 °C) or decreased (4 °C) ambient temperatures or to ethanol-induced hypothermia [13], which was confirmed using the Thermal Gradient Ring [14], [15]. Body temperature elevation in response to the bacterial pyrogen lipopolysaccharide (LPS) was significantly attenuated in TRPV1KO mice; however, there were no significant differences between WT and TRPV1KO mice in the extent of LPS-induced c-Fos expression in numerous fever-related brain subregions. In another study, TRPV1KO mice were hypometabolic with lower oxygen consumption, showed hypervasoconstriction with lower tail skin temperature, and had a higher thermoneutral zone [16]. TRPV1KO mice also preferred a lower ambient temperature, which was not confirmed using the Thermal Gradient Ring [14], [15], but had higher locomotor activity, which was consistent with research using the Thermal Gradient Ring [14], [15]. Overall, TRPV1KO mice look showing a little different thermoregulatory phenotype compared with WT mice, which may be coupled with a predisposition to age-associated weight gain, including hypometabolism, enhanced vasoconstriction, decreased thermopreferendum, and hyperkinesis. However, it is not clear whether those phenotypes of TRPV1KO mice are related to the thermosensing ability of TRPV1.

It was also reported that TRPV1KO mice exhibit transient hyperthermia when exposed to 30.0–32.5 °C, whereas WT mice do not [17]. TRPV1KO mice exhibited prolonged and prominent hyperthermia upon exposure to 35.0 °C, while WT mice showed transient hyperthermia. Hyperthermia also occurred in WT mice that received intracerebroventricular injections of the TRPV1 antagonist AMG9810 upon exposure to 35.0 °C. These results indicate that central TRPV1 is critical for maintaining a constant body temperature via heat loss behavior under warm ambient temperatures, and this is consistent with a report of TRPV1 expression in brain [18]. However, more investigation is needed.

## 5 TRPV1 antagonists

After the initial cloning of TRPV1, several pharmaceutical companies worldwide attempted to develop TRPV1 antagonists because of their role as excellent pain killers. However, there is currently no TRPV1 antagonist on the market partly because many of these antagonists induced severe hyperthermia sometimes over 39 °C in both animal models and human clinical trials. For example, oral administration of AMG517 increased the body temperature of some participants to 39–40 °C for 1–4 days [19]. Other TRPV1 antagonists (e.g., ABT-102, AZD1386, and JNJ-39439335) also produced varying degrees of hyperthermia at therapeutic dosages [20], [21], [22]. However, the action sites of the chemicals and mechanism of action underlying these thermoregulatory effects of TRPV1-selective antagonists are poorly understood. Hyperthermia is not observed in the systemic TRPV1 KO mice, but occurs with acute inhibition in WT mice treated with TRPV1 antagonists. One possible explanation for this result is that systemic TRPV1 KO mice, which lack TRPV1 function from the embryonic stage through postnatal development, may have a compensatory thermoregulatory system to prevent TRPV1-deficiency-induced thermoregulatory defects, and acute inhibition of TRPV1 function causes significant thermoregulatory effects. Inhibition of TRPV1 function in the innervating vasculature of peripheral neurons may modulate vascular tone, but not enough to cause systemic body temperature changes.

Interestingly, the Romanovsky group found that the TRPV1 antagonists A-1165901 and AMG7905 caused hypothermia, instead of hyperthermia, in rodents [23]. *In vitro*, both A-1165901 and AMG7905 potentiated TRPV1 activation by protons while blocking TRPV1 activation by capsaicin. The authors discussed that TRPV1 is tonically activated by protons and drives the reflectory inhibition of thermogenesis and tail-skin vasoconstriction. That work suggested that both side effects (hypothermia and hyperthermia) can be dealt with simultaneously, by minimizing the drugs’ interference with TRPV1 activation by protons [23]. The same group reported that this mechanism starts with TRPV1 in skeletal muscles of the trunk [24].

The Julius group attempted to address the above by selectively eliminating TRPV1 expression in sensory neurons or vascular smooth muscle cells; they found that elimination of TRPV1 in sensory neurons abrogated agonist-induced hypothermia and antagonist-induced hyperthermia [25]. Furthermore, lesioning of the central projections of TRPV1-positive sensory nerve fibers abrogated drug-mediated thermoregulation. Thus, they concluded that TRPV1 drugs alter core body temperature by modulating sensory input to the central nervous system. This conclusion suggests that mechanically distinct TRPV1 antagonists may diminish inflammatory pain without affecting core body temperature, and supports the idea that different signaling pathways work upon activation of the same protein TRPV1 as reported for TRPA1 [26].

Recently, by examining the effects of several different TRPV1 antagonists on body temperature, the Yu group reported that the impact of TRPV1 analgesics on proton gating does not disturb core body temperature [27]. After comparing the cryo-EM-based structures with TRPV1 antagonists, they concluded that TRPV1 antagonists cause hyperthermia by acting on the TRP box; however, it is still not clear how the TRP box is involved in hyperthermia.

## 6 Structural basis for heat-evoked activation of TRPV1

Regarding the structural determinant(s) of heat-evoked activation, the contributions of distinct regions of TRPV1 have been reported, as follows: deletion of the C-terminus lowered the temperature thresholds for heat-evoked activation [28], [29], [30]; the membrane-proximal domain connecting N-terminal ankyrin repeats and the first transmembrane domain determined temperature dependence [31]; heat activation and shifts in threshold temperature are intrinsic to the pore domain [32], [33]; and the ankyrin repeat domain is important for heat-evoked activation [34], [35]. These results suggest that structural determinants of heat-dependent activation are spread over the TRPV1 molecule. However, primary modules determining the temperature thresholds of TRPV1 remain unclear. In this regard, a recent work showed that two amino acids (Gln, Leu/Val) in the ankyrin repeat 1 of the N-terminus are conserved among tailed amphibians with low temperature thresholds but are different from those (Arg, Lys) in rat TRPV1 [36], suggesting that the ankyrin repeat domain may function as a structural module contributing to temperature sensitivity control. This is consistent with a recent report that two charged residues in the ankyrin repeat domain of mosquito TRPA1 determine the temperature thresholds for heat-evoked activation [37], suggesting that this is widely applicable for thermosensitive TRP channels.

## 7 Cryo-EM structure of TRPV1 and heat-evoked activation

**Fig. 4:**
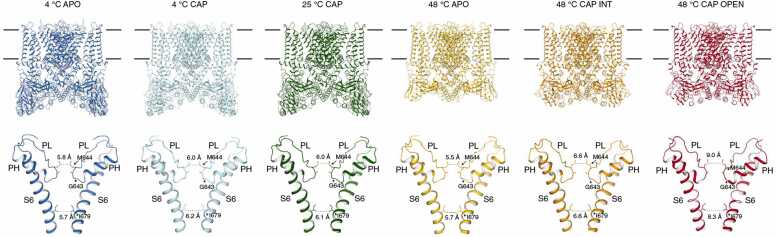
Structures of TRPV1 under six conditions (4 °C APO (without capsaicin binding), 4 °C capsaicin (CAP), 25 °C Cap, 48 °C APO, 48 °C intermediate (INT), and 48 °C Open). Gradual pore dilation was observed (modified from Kwon et al., 2021).

The structure of TRPV1 was clarified by single-particle analysis using cryo-EM at the atomic level [38], [39], and also using lipids (nanodisc) [40]. A higher-resolution structure of TRPV1 was recently reported by the same group, and the mechanism of polymodal functionality was clarified [41]. Noxious heat-dependent (temperatures at 4, 25 and 48 °C) TRPV1 opening comprised stepwise conformational transitions in the presence of capsaicin [42]. Global conformational changes across multiple subdomains of TRPV1 are followed by rearrangement of the outer pore, leading to gate opening; this suggests that each distinct domain is involved in the response to heat stimulation, and that mutations affecting heat sensing or coupling mechanisms cannot be functionally distinguishable (Fig. 4). However, it is still not clear how heat stimulus initially affects the TRPV1 structure.

## 8 Thermodynamics of TRPV1 activation

As mentioned above, the temperature-sensing mechanism of TRPV1 has not been clarified. Thermodynamics dictate that TRPV1 must undergo an unusually energetic allosteric transition. Thus, it is important to directly measure the energetics of this transition for proper determination of the temperature-sensing mechanism [43]. In this regard, Voets et al. introduced the principle of temperature-dependent gating in TRPV1 and proposed that temperature sensing is tightly linked to voltage-dependent gating [44]. According to this principle, TRPV1 is activated by depolarization, and changes in temperature result in a graded shift in the voltage-dependent curve. Capsaicin is thought to function as a gating modifier, shifting the activation curve towards physiological membrane potentials. Kinetic analysis of gating at different temperatures indicates that the temperature sensitivity of TRPV1 arises from a 10-fold difference in the activation energy associated with voltage-dependent opening and closing. Although this principle can be applied to the cold-evoked activation of TRPM8, there have been no subsequent works.

Consistent with this concept, Clapham and Miller claimed that the laws of thermodynamics dictate that opening of TRPV1 must involve an unusually large conformational standard-state positive enthalpy, which recalls long-appreciated principles of protein folding [45]. They also suggest that TRPV1 gating is accompanied by large changes in the molar heat capacity.

## 9 Evolution of TRPV1

Rodents have 11 thermosensitive TRP channels, with four belonging to the TRPV subfamily (TRPV1-TRPV4), five to the TRPM subfamily (TRPM2-TRPM5, and TRPM8), one to the TRPA subfamily (TRPA1), and one to the TRPC subfamily (TRPC5) [46]. Thermal perception by insect species such as fruit flies involves two channels belonging to the TRPC subfamily (TRP and TRPL), three to the TRPA subfamily (TRPA1, painless, and pyrexia), and three to the TRPP subfamily (biribido-1, −2, and −3), although whether all of these proteins function as ion channels is unclear [47]. TRP channels involved in thermal perception largely differ between invertebrates and vertebrates.

In addition to TRPA1, vertebrate species possess another heat-sensitive channel, TRPV1, which likely emerged in a common ancestor of tetrapods and teleosts. TRPV1 is activated by heat, a feature consistent across various vertebrate species, including rodents, chickens, snakes, amphibians, and teleosts. TRPV1 is often co-expressed with TRPA1 in subsets of DRG and trigeminal ganglion (TG) neurons in vertebrate species such as rodents, chickens, clawed frogs, and zebrafish, suggesting that TRPV1 was likely acquired as a heat-sensitive channel in the most recent common ancestor of tetrapods and teleosts, leading to redundancy among heat receptors.

Infrared detection has been acquired in vertebrates. Vampire bats are obligate blood feeders that locate hotspots on endothermic prey by detecting infrared radiation using leaf pits surrounding the nose. Heat-sensitive neurons innervating leaf pits respond to temperatures above 29 °C. Infrared detection by vampire bats is thought to involve TRPV1, which is expressed as two alternatively spliced (AS) variants in these bats. In the novel AS variant termed TRPV1-S, the C-terminal region is truncated relative to the canonical AS variant termed TRPV1-L. TRPV1-S is predominately expressed in the TG and is activated by temperatures around 30 °C, whereas TRPV1-L is expressed in the DRG and is activated by temperatures around 40 °C. Therefore, vampire bats likely rely on this TRPV1-S variant with higher thermal sensitivity for infrared detection [30].

The ability to sense heat is crucial for survival. Increased heat tolerance may prove beneficial by conferring an ability to inhabit otherwise prohibitive ecological niches. Ground squirrels (in the intermittent arousal during hibernation) and camels (in the hot ambient temperatures of deserts) can tolerate temperatures exceeding 40 °C better than many other mammalian species can. TRPV1 of ground squirrels and camels is less activated by high temperatures [34]. This decreased thermosensitivity is an evolutionary adaptation targeting a focused aspect of TRPV1 function in the detection of noxious heat through the skin, sparing its role in nonthermal aspects of nociception, inflammation, and thermoregulation.

## 10 TRPV1 mutation in humans

Two affected individuals carrying a homozygous missense mutation in TRPV1 that renders the channel nonfunctional have been reported [48]. The affected individuals were not sensitive to application of capsaicin to the mouth or skin. The examination revealed an elevated heat-pain threshold but also, surprisingly, an elevated cold-pain threshold, although the mechanism is unknown.

Malignant hyperthermia is a pharmacogenetic disorder arising from uncontrolled muscle Ca^2+^ release due to an abnormality in the mechanism of sarcoplasmic reticulum Ca^2+^ release triggered by halogenated inhalational anesthetics. Although this pathology may be linked mainly to mutations in the ryanodine receptor gene (RyR1), *TRPV1* variants have been reported in two patients [49], [50]. The mutant TRPV1 was shown to be activated by isoflurane. However, further investigation is needed.

As stated above, TRPA1 and TRPM3 were also found to be reported to detect noxious heat stimuli because mice with triple KO of TRPV1, TRPM3 and TRPA1 completely lost their response to noxious heat stimuli [11]. However, in a human randomized crossover trial with specific antagonists (BCTC for TRPV1; A-967079 for TRPA1; Naringenin for TRPM3), only TRPV1 involvement in heat-evoked pain sensation was confirmed [51].

## Perspective

We still know very little about the function of TRPV1. More studies are needed especially regarding the thermosensing and thermoregulatory mechanisms of TRPV1, which could shed light on global warming issues.

## Consent to participate

Consent to participate are not applicable for the data shown in this review.

## CRediT authorship contribution statement

**Tominaga Makoto:** Funding acquisition, Writing – original draft, Writing – review & editing. **Iwata Moe:** Data curation.

## Declaration of Competing Interest

All authors declare that no support, financial or otherwise, has been received from any organization that may have an interest in the submitted work.

## Data Availability

All data and materials used in the analysis are available in the manuscript or the cited works by the authors.
